# Characterization of *CcTFL1* Governing Plant Architecture in Pigeon pea (*Cajanus cajan* (L.) Millsp.)

**DOI:** 10.3390/plants12112168

**Published:** 2023-05-30

**Authors:** Isha Mendapara, Kaushal Modha, Sunayan Patel, Vipulkumar Parekh, Ritesh Patel, Digvijay Chauhan, Kirti Bardhan, Manzer H. Siddiqui, Saud Alamri, Md Atikur Rahman

**Affiliations:** 1 Department of Genetics and Plant Breeding, N. M. College of Agriculture, Navsari Agricultural University, Navsari 396 450, Gujarat, India; ikmendapara@gmail.com (I.M.); riteshpatel@nau.in (R.P.); 2 Department of Genetics and Plant Breeding, College of Agriculture, Navsari Agricultural University Campus, Bharuch 392 012, Gujarat, India; srpatel@nau.in; 3 Department of Basic Science and Humanities, College of Forestry, Navsari Agricultural University, Navsari 396 450, Gujarat, India; vipulbiotech@nau.in (V.P.); kirtivardhan@nau.in (K.B.); 4 Pulses and Castor Research Station, Navsari Agricultural University, Navsari 396 450, Gujarat, India; dachauhan@nau.in; 5 Department of Botany and Microbiology, College of Science, King Saud University, Riyadh 11451, Saudi Arabia; mhsiddiqui@ksu.edu.sa (M.H.S.); saualamri@ksu.edu.sa (S.A.); 6 Grassland & Forage Division, National Institute of Animal Science, Rural Development Administration, Cheonan 31000, Republic of Korea; atikbt@korea.kr

**Keywords:** terminal flowering locus, growth habit, allelic characterization, InDel and motif

## Abstract

Growth habits are among the essential adaptive traits acted upon by evolution during plant speciation. They have brought remarkable changes in the morphology and physiology of plants. Inflorescence architecture varies greatly between wild relatives and cultivars of pigeon pea. The present study isolated the *CcTFL1* (*Terminal Flowering Locus 1*) locus using six varieties showing determinate (DT) and indeterminate (IDT) growth habits. Multiple alignments of *CcTFL1* sequences revealed the presence of InDel, which describes a 10 bp deletion in DT varieties. At the same time, IDT varieties showed no deletion. InDel altered the translation start point in DT varieties, resulting in the shortening of exon 1. This InDel was validated in ten varieties of cultivated species and three wild relatives differing in growth habits. The predicted protein structure showed the absence of 27 amino acids in DT varieties, which was reflected in mutant CcTFL1 by the absence of two α-helices, a connecting loop, and shortened β-sheet. By subsequent motif analysis, it was found that the wild-type protein had a phosphorylation site for protein kinase C, but the mutant protein did not. In silico analysis revealed that the InDel-driven deletion of amino acids spans, containing a phosphorylation site for kinase protein, may have resulted in the non-functionality of the CcTFL1 protein, rendering the determinate growth habit. This characterization of the *CcTFL1* locus could be used to modulate growth habits through genome editing.

## 1. Introduction

Pigeon pea [*Cajanus cajan* (L.) Millsp.] is a major food legume crop grown primarily in tropical and subtropical regions of the world. It is cultivated in ~5 million hectares worldwide and provides dietary protein to more than 1 billion people. Additionally, it provides income to millions of resource-poor farmers throughout Asia, Africa, the Caribbean, South America, and Central America [1].

The changes in crop plant morphology and physiology, such as inflorescence architecture and photoperiodism, could be the result of evolution in conjunction with domestication or selection. During the evolution of various flowering plants, these two adaptive traits have been subjected to intense natural selection. Different genes, their interactions, and external environmental cues, such as photoperiod, light quality, and temperature, all influence shoot architecture and floral transition [2,3]. However, several interconnected pathways govern floral transition, including photoperiodic, vernalization, ambient temperature, and autonomous and hormonal signaling [3,4]. In general, a linkage is observed between crop growth habits and the photoperiodic response of flowering time. In common beans, the repulsion phase of linkage was observed between the recessive determinacy locus (*fin*) and dominant photoperiod sensitivity locus (*Ppd*) in the D1 linkage group [5]. In lablab beans, a coupling phase of linkage was observed between growth habit and photoperiod responsive flowering [6,7].

*Flowering Locus T* (*FT*) and *Terminal Flower Locus* (*TFL*) are two important loci that control flowering time and stem architecture [4]. The *FT* and *TFL* genes encode small proteins that are members of the phosphatidyl ethanolamine-binding protein (PEBP) family. The members of this gene family play an important role in the growth and differentiation process of many plants, animals, yeast, and bacteria by regulating various signaling pathways [3,4,8]. *FT* and *TFL* homologs share nearly 60% sequence identity but have opposing functions [8]. *FT* promotes the transition of shoot apical meristem (SAM) into the reproductive bud, whereas *TFL* inhibits this transition and thus acts as a negative regulator of flowering. In vivo gain-of-function studies revealed distinct phenotypes, indicating that protein sequence, rather than expression profiles, determine antagonistic functions of *TFL1* and *FT* homologs [3].

The growth habit can differ considerably among cultivars for a given crop. Some cultivars have a vegetative terminal bud, allowing the genotype to grow tall and spread under favorable conditions; this is known as an indeterminate habit. The other form of growth habit is the determinate type, in which the main shoot axis stops vegetative growth and terminates in a floral structure. In general, wild relatives of pigeon pea exhibits IDT growth habit. The determinate varieties mature rapidly and have a shortened flowering period, allowing for early maturity and easy mechanical harvesting. A single recessive gene was found to control growth habits in pigeon pea [9].

In Arabidopsis, TFL1 caused indeterminacy along with delayed floral transition [10]. The TFL homologue SP (SELF PRUNING) was identified to influence tomato growth habit [11]. TFL1 homologs have been identified in a variety of legumes, including common beans, cowpea, pea, and soybean [12]. In peas, two homologous loci were identified: PvTFL1a as the Determinate (DET) gene and PvTFL1c as the Late Flowering (LF) gene [13]. Previous studies of common bean found that PvTFL1y is responsible for naturally occurring variation in determinacy, co-segregated with the determinacy locus *fin*, and is the functional homologue of Arabidopsis TFL1 [14]. A TFL1 homologue governing the indeterminate growth habits of Arabidopsis was identified; its transcriptional repression was shown to delay flowering transition [15]. *CcTFL1* was identified as a candidate gene for determinacy in pigeon pea by QTL analysis [16,17]. Continuous flowering was observed in roses and strawberries due to recessive homologs of *TFL*, *RoKSN*, and *FvKSN*, respectively [18]. These findings indicated that *TFL* homologue is responsible for variation in growth habits in pulses as well as diverse plant species, indicating the conserved function of TFL.

The *TFL* locus has been characterized in many pulses, but its molecular function remains enigmatic. However, marker-assisted selection for growth habits in pigeon pea has been reported using SNP and InDel [16,17]. *TFL* participates in complex flowering pathways, in addition to its role in growth habit regulation. Given the recent trends in genome editing and synthetic biology, complete gene characterizations are among the most important prerequisites for redesigning organisms with expected characteristics that can be used in directed evolution. The absence of well-characterized genomic regions for the preferred function is a setback in the synthesis of desired biological networks or whole biological systems [19]. Looking at the lack of information on the complete characterization of *CcTFL1* in the literature and in databases, an effort was made in the current study to fully characterize this gene. In conjunction with protein functional studies, gene characterization may provide better insights into growth habit control and aid in deciphering the complex flowering pathways in pigeon pea.

## 2. Results

### 2.1. PCR Amplification and Allelic Characterization of CcTFL1

**Figure 1:**
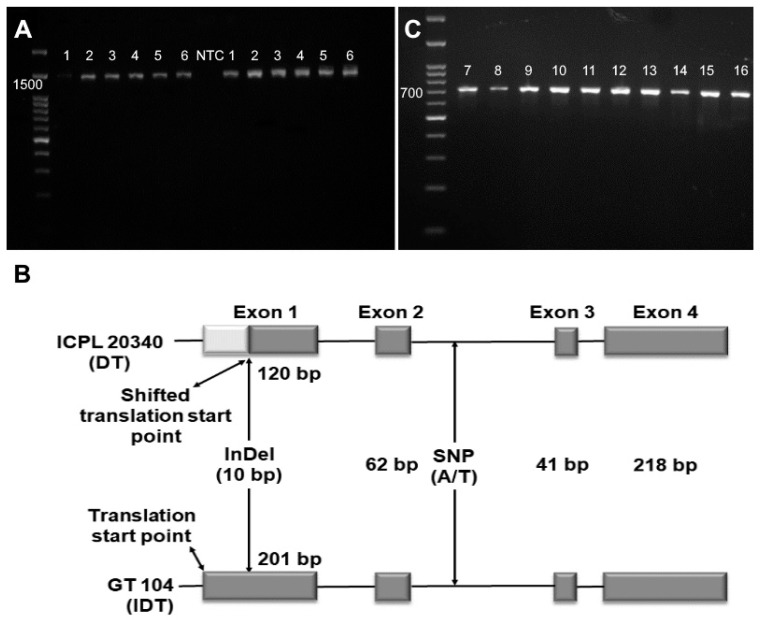
*CcTFL1* locus amplification and characterization. (**A**) Agarose gel image of a 1400bp amplified complete gene sequence in six varieties. M: 100bp ladder; PADT 16, ICPL 20340, BDN 711, Vaishali, GT 104, and GT 105 (two replicas); NTC: Non-template control. (**B**) *CcTFL1* gene structure in pigeon pea. Exon 1 of GT 104 is 201 bp long. Due to InDel, ICPL 20340 has an 81-bp shorter exon 1 than the IDT variety. (**C**) Agarose gel image of a gene sequence of 700 bp in 10 varieties for InDel and SNP validation. 7-16 lane: GT 100, ICPL 87, AVPP 1, ICPL 20336, ICPL 11258, GT 103, GT 101, P 992, AGT 2, and UPAS 120, respectively.

The gene sequence (~1500 bp) was amplified using the primers listed in Table 1 (Z505 and Z506). The amplicons (~1500 bp) were sequenced from all six varieties (Figure 1A). With a 6e^−168^ E-value, BLASTn analysis revealed the highest sequence identity of 82.62% with the *Glycine max Dt1* gene, a homologue of *TFL* locus. The obtained sequences were also similar to *PvTFL1y*, *LprTFL*, *GsDt1,* and *VuTFL1* loci, with 81.16%, 79.63%, 78.90%, and 78.90% identity, respectively.

**Figure 2:**
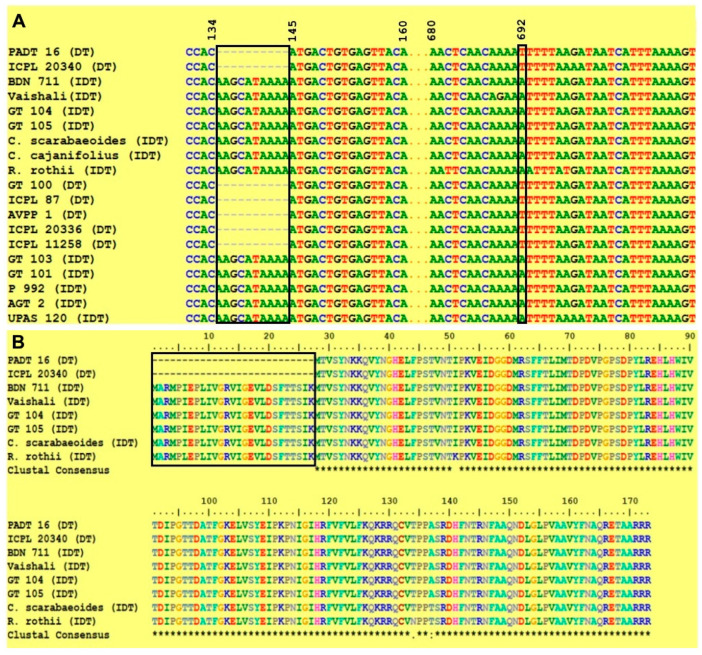
Multiple sequence alignment of pigeon pea genotypes. (**A**) Multiple sequence alignment of *TFL1* gene sequence from 16 *C. cajan* varieties, *C. scarabaeoides*, *C. cajanifolius* and *R. rothii*. Rectangles show identified functional InDel markers (135–144 bp) and SNP (692 bp). (**B**) Multiple sequence alignments of TFL1 protein sequence from six *C. cajan* varieties as well as two wild relatives of pigeon pea (*C. scarabaeoides* and *R. rothii*). The rectangle shows a deleted span of 27 amino acids in two determinate varieties, i.e., PADT 16 and ICPL 20340, due to the shifting of the translation start point owing to the presence of InDel.

Multiple sequence alignment using BioEdit CLUSTALW revealed 10 bp deletion in the initial sequence of determinate varieties (Figure 2A). Except for the first exon, all exon predictions by GeneMark were found to be identical (Figure 1B). The start point and the length of first exon varied between both the alleles (Table 2). Because of an InDel-driven alteration in the translation start site, all DT varieties had a 120 bp first exon. In contrast to DT varieties, IDT varieties displayed the first exon of 201 bp length, with an average start point of 81 bp earlier. Furthermore, a previously reported SNP was found at the 692nd position within an intronic region by GeneMark analysis (Figure 2A).

### 2.2. Validation of InDel

Apart from the six varieties used for complete gene characterization, a total of ten varieties were used to validate the InDel identified in *CcTFL1*. A 700 bp amplicon was obtained after amplifying a partial sequence containing InDel (Figure 1C). These amplicons were sequenced and subjected to multiple sequence alignment. The findings revealed that DT varieties had a 10 bp deletion in exon 1. In contrast, IDT varieties revealed no such deletion. This finding indicated a link between InDel polymorphism and growth habits in pigeon pea. The complete *CcTFL1* sequence was amplified in two samples (*Cajanus scarabaeoides* and *Rhynchosia rothii*), and partial amplification was achieved in one sample (*Cajnanus cajanifolius*), while *Rhynchosia minima* and *Canavalia gladiate* showed no amplification. However, the *CcTFL1* gene sequence obtained from wild relatives corresponded to the sequence of IDT varieties with a 10 bp insertion.

### 2.3. Protein Modeling and Structural Comparison

**Figure 3:**
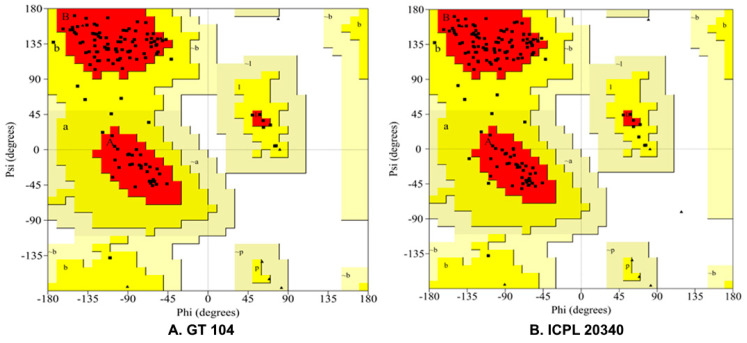
Ramachandran plots of modeled protein structures for pigeon pea CcTFL1, constructed using PROCHECK. (**A**) GT 104 CcTFL1 protein structure enclosed 89.9% non−glycine residues in a most favored region, with the remaining 10.1% in additional allowed regions. (**B**) The CcTFL1 protein structure of ICPL 20340 encompassed 89.0% of non−glycine amino acid residues in the most favored regions, with the remaining 11.0% in additionally allowed regions. Neither generously allowed nor disallowed regions contained a single amino acid residue from either protein. Red colored segments (A, B and L) represent the most favored regions. Yellow segments (a, b, l and p) covers additional allowed regions. Beige colored sections [~a, ~b, ~l, ~p] represent generously allowed regions. Glycine residues are shown as triangles; whereas, non-glycine amino acid residues are represented as squares.

The absence of 27 amino acids (1–27) in PADT 16 and ICPL 20340 was discovered through multiple alignments of translated protein sequences from all six varieties. These amino acids, in contrast, were found in BDN 711, Vaishali, GT 104, and GT 105, as well as two wild relatives of pigeon pea. (Figure 2B). The SWISS-MODEL server was used to predict the structure of CcTFL1 protein sequences from ICPL 20340 (DT) and GT 104 (IDT). The reliability of the modeled protein structures was confirmed by GMQE (ICPL 20340: 0.86 and GT 104: 0.87) and QMEAN Z score (ICPL 20340: −0.15 and GT 104: −0.49). In the CcTFL1 protein structure of ICPL 20340, Ramachandran plot analysis using the PROCHECK server revealed that 89.0% of the non-glycine amino acid residues were contained in the most preferred regions, with 11.0% residues in additionally allowed regions (Figure 3). The GT 104 CcTFL1 protein structure contained 89.9% non-glycine residues in the most preferred region, with the remaining 10.1% in other allowed regions. Neither the allowed nor the forbidden region contained a single amino acid residue.

**Figure 4:**
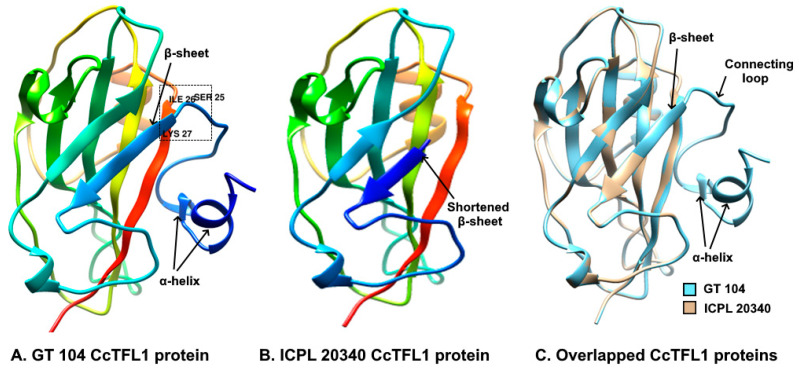
Comparison of predicted CcTFL1 protein structures generated by homology modeling with SWISS-MODEL server. (**A**) 3D structure of predicted wild type CcTFL1 protein in GT 104. (**B**) 3D structure of predicted mutant CcTFL1 protein in ICPL 20340. (**C**) Overlapped 3D protein structures of both wild-type and mutant CcTFL1 proteins. The box represents the motif site in wild-type protein containing ‘SIK’ amino acid residues (25–27), absent in mutant CcTFL1 protein. Colored ribbons with arrow head represent β-sheets. Colored coils represent α-helices and colored cylindrical riboon represents connecting loops.

UCSF Chimera was used to visualize the modeled structures. It was discovered that the first 27 amino acids are responsible for the formation of two α-helices, a connecting loop, and a portion of a β-sheet in GT 104. The deletion of these 27 amino acids in ICPL 20340 resulted in the complete loss of the two α-helices and the connecting loop and a shortening of the β-sheet, resulting in a structural anomaly of the CcTFL1 protein of the mutant allele (Figure 4).

### 2.4. Sequence Motif Discovery

**Figure 5:**
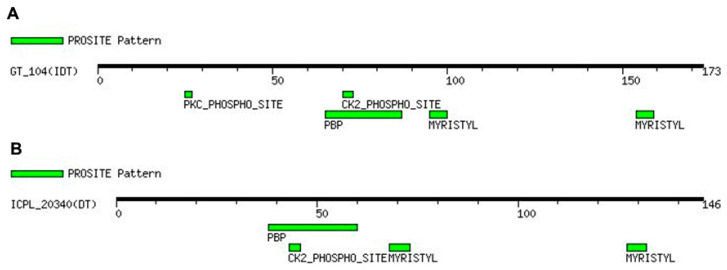
Identified motifs and their location across the protein sequence. (**A**) Motifs identified in protein sequence of GT 104 (IDT) variety. (**B**) Motifs present in the protein sequence of the ICPL 20340 (DT) variety. Four motifs are present in the wild type protein sequence of GT 104, whereas only three motifs exist in ICPL 20340, illustrating the absence of the PKC_PHOSPHO_SITE motif.

Using a protein query sequence against the PROSITE database, we discovered four distinct motifs in the GT 104 protein sequence: MYRISTYL, PBP, CK2 PHOSPHO SITE, and PKC PHOSPHO SITE (Figure 5A). In contrast, only three motifs were discovered in the protein sequence of ICPL 20340: MYRISTYL, PBP, and CK2 PHOSPHO SITE (Figure 5B). The PBP motif is a phosphatidyl ethanolamine-binding protein family signature found in both proteins. In the wild-type protein, the Protein kinase C phosphorylation site (PKC PHOSPHO SITE) motif encloses ‘SIK’ amino acid residues at positions 25–27, which form a part of the connecting loop, as well as the β-sheet. The absence of a phosphorylation site in mutant CcTFL1 protein was caused by the deletion of these amino acid residues.

## 3. Discussion

Photoperiods and growth habits have influenced the evolution and domestication of many pulse crops. The majority of wild relatives, landraces, and cultivars of grain legumes, including pigeon pea, are of the indeterminate type, demonstrating the prevalence of this growth habit in nature. However, some cultivars exhibit determinate growth habits, with bushy plant types with fewer branches and nodes, lodging resistance, fewer pods with higher seed weight, photo-insensitivity, early maturity, and feasibility of mechanized harvesting due to synchronized maturity. These characteristics make determinate growth habits more desirable for most grain legumes, including pigeon pea.

Efforts have been made to understand the genetic basis of determinate growth habits in Arabidopsis [10,20], as well as in many legume crops [13,21,22]. Legumes share synteny among their genomic regions, indicating a higher level of conserved gene sequence, particularly in the Phaseoloid clade [16]. Furthermore, the availability of genome sequences facilitates analyses of gene function and genetic networks. The TFL1 locus causes a delay in flower transition as well as shoot inflorescence indeterminacy in Arabidopsis [10]. Two different homologs of *TFL1*, i.e., *DET* and *LF*, are responsible for determinate growth habits and delayed flowering in *Pisum sativum* [13]. *PvTFL1y* co-segregates with the *fin* locus and is responsible for growth habit phenotypes. It is also a functional homologue of *Arabidopsis TFL1* [14,21]. In *Glycine max*, determinacy is caused by a recessive allele of the *Dt1* locus, which is an ortholog of *Arabidopsis TERMINAL FLOWER 1* [2,23]. *CcTFL1* has been identified as a possible candidate gene for determinacy in pigeon pea [16]. The characterization of *LprTFL* resolved the difference in growth habits in Indian beans (*Lablab purpureus*) [22]. *MiTFL1* has been identified as a negative flowering regulator in mango [24].

The *CcTFL1* locus was successfully amplified from DT and IDT varieties of cultivated/wild relatives in this study (Figure 1A,C). The characterization of *CcTFL1* indicated that an InDel at the start point of the first exon is associated with a change in growth habits in pigeon pea. This InDel caused the deletion of the initiation codon, causing the shift in translation start point of the first exon to 81 bp downstream in DT varieties as compared to cultivated IDT varieties/wild relatives (Figure 1B). This eventually resulted in the shortening of first exon in determinate varieties. Additionally, one SNP (A/T) was also found to be present in the intronic region (Figure 1B). SNP allele ‘A’ was present in all DT varieties, whereas all IDT varieties showed the presence of the ‘T’ allele, illustrating the strong association of SNP with growth habits in pigeon pea and its wild relatives. The same SNP (A/T) was identified in the *CcTFL1* gene, which enabled discrimination of all DT lines from the IDT lines using PCR-based SNP assay [16]. Functional annotation of 10 bp InDel in *CcTFL1* confirmed frameshift mutation in determinate varieties [17].

The loss of 14 amino acids in mutant TFL protein driven by splice site SNP at the endpoint of the third exon was responsible for the transformation of shoot apical meristem (SAM) into flower bud in the determinate genotype of *Lablab purpureus* [22]. In pea, sequencing of *PsTFL1a* in three independent determinate mutant lines revealed synonymous or nonsynonymous substitution at the exon–intron junction after the third exon, resulting in non-functionality of TFL protein due to splicing failure [13]. An association of two haplotypes, a retrotransposon and a splice site alteration, was observed with determinacy at the *PvTFL1y* locus in common bean [21]. A sequence variation analysis of the *VuTFL1* homologue revealed transversion of C to A in exon 4, which led to a change in amino acid, affecting protein function and stability in determinate mutants of cowpea [25]. Four distinct nonsynonymous substitutions were observed in the *GmTFL* locus in cultivated soybean genotypes. Each one of them impelled the transition of meristem identity from indeterminate to determinate type [23].

An in silico analysis in the present study revealed an InDel driven loss of 27 amino acids in the CcTFL1 protein sequence of DT varieties due to a shift in the translation start point (Figure 2B). This N-terminal truncated mutant CcTFL1 protein might remain in the cytoplasm due to loss of the N-terminal signal peptide required for entering the nucleus. The deleted amino acid span harbored the PKC_PHOSPHO_SITE motif at the 25–27 position (SIK), which is the site required for phosphorylation by protein kinase C (Figure 5). This phosphorylation site was observed in the wild-type protein sequence of CcTFL1. Protein kinase C exhibits a preference for the phosphorylation of serine and threonine amino acid residues, which are present near the C-terminal [26]. In the present scenario, the discovered motif represents the ‘SIK’ amino acid sequence, evidencing the specificity of protein kinase C for serine (Figure 4A). The phosphorylation of protein is significantly involved in post-translational modifications [27,28], protein regulation [27,28], protein crosstalk [11,28,29], protein distribution ratio, and subcellular localization [28,30]. *TFL* interacts with bZIP transcription factor *FD*, forming the TFL-FD complex to suppress flowering by inhibiting floral-inducing genes, e.g., *AP1* [15,29]. In contrast, *FT* interacts with *FD*, forming an FT-FD complex and resulting in floral induction. The phosphorylation of threonine residue by protein kinase was found to be crucial for transcription factor *FD* to form a complex with PEBP family protein, i.e., *FT* [29]. *TFL1* is known as a mobile transcription co-factor, and its movement is crucial for regulating meristem indeterminacy [31]. Phosphorylation activity and the identification of protein kinase are crucial to comprehend florigen activity at SAM [32]. As a transcription co-factor, the interaction of *CcTFL1* with other proteins, as well as its subcellular localization and protein signaling, can be dependent on phosphorylation by protein kinase C. The inability of kinase to phosphorylate mutant CcTFL1 protein due to the absence of the PKC_PHOSPHO_SITE motif might be responsible for determinate growth habit. These in silico findings are to be further validated by empirical experiments.

## 4. Materials and Methods

### 4.1. Plant Materials and Phenotyping

*CcTFL1* allelic characterization was performed on six pigeon pea varieties with extreme phenotypes in terms of growth habits. ICPL 20340 and PADT 16 were classified as having determinate growth habits because they demonstrated the transition of the shoot apical meristem into reproductive architecture. In varieties Vaishali, GT 104, GT 105, and BDN 711, the terminal bud continued to grow vegetatively and did not show floral transition at the shoot apex. As a result, these four varieties were labeled as indeterminate. For allelic variation validation, ten varieties were chosen, including determinate (GT 100, ICPL 87, AVPP 1, ICPL 20336, and ICPL 11258) and indeterminate (GT 103, GT 101, P 992, AGT 2, and UPAS 120) types. Five wild accessions with indeterminate growth habits (*Cajanus cajanifolius, Cajanus scarabaeoides, Rhynchosia minima, Rhynchosia rothii,* and *Canavalia gladiate*) were also included for broader validation. The varieties were all grown in normal field conditions under short-day conditions.

### 4.2. Primer Designing and PCR Amplification

The *Cajanus cajan* whole genome shotgun sequence (variety Asha; GenBank accession: NW017984051.1) was used as a template for designing primers to amplify the entire gene sequence of *CcTFL1* using the NCBI primer designing tool Primer-BLAST. These designed primers were expected to amplify the entire 1500 bp *CcTFL1* gene sequence (Table 1). Using the cetyl trimethyl ammonium bromide (CTAB) method [33], genomic DNA was isolated from young trifoliate leaves. To amplify the intended locus, polymerase chain reaction (PCR) was performed using GT-PCR Master Mix (TaKaRa, Clontech, Japan). The PCR reaction was set up in a 20 µL final volume with 200 ng genomic DNA, 10 µL master mix, and 10 pmol forward and reverse primers. The PCR protocol included 7 min of initial denaturation at 95 °C, 35 cycles of denaturation for 30 s at 94 °C, annealing for 45 s at 63 °C, an extension for 45 s at 72 °C, and a final extension for 10 min at 72 °C. On 1.5% agarose gel electrophoresis, the PCR products were resolved.

### 4.3. Sequencing and Characterization of CcTFL1

The amplified genes from six varieties were subjected to bidirectional Sanger sequencing with gene-specific primers on an ABI 3730xl Genetic Analyzer using a BDT v3.1 Cycle sequencing kit. The reverse primer nucleotide sequence was converted into a reverse complementary sequence. A consensus sequence was created by identifying an overlapping sequence between forward and reverse complementary sequences. The GT 104 consensus sequence was used as a query sequence in NCBI-BLASTn analysis [34]. Furthermore, to determine sequence polymorphism, all sequences were subjected to CLUSTALW multiple sequence alignment [35]. For gene prediction, Eukaryotic GeneMark.hmm version 3.54 [36] was used to locate exons and introns in sequences of reasonable length. Open reading frames (ORFs) of all six varieties were created by joining all exons and then translated to predict protein sequences with BioEdit [35]. These sequences have been submitted to the NCBI under the accession numbers ON711024 to ON711029 (Supplementary Material).

### 4.4. Validation of Allelic Variation

To validate allelic variation, genomic DNA was isolated from young trifoliate leaves of 10 varieties (five each of DT and IDT) and five wild relatives. The *CcTFL1* locus sequence containing InDel was amplified using the primers CcTFL1_f5b_F and CcTFL1_f5a_R (Table 1). The PCR reaction mix and cycle setup remained the same as previously described. The amplicons were sequenced, and then multiple sequence alignment was performed with the BioEdit CLUSTALW multiple sequence alignment tool [35]. These sequences have been submitted to the NCBI under the accession numbers ON711014 to ON711023, OQ540751, OQ540752, and OQ540753 (Supplementary Material).

### 4.5. Protein Homology Modeling and Motif Analysis

The predicted protein sequences of GT 104 and ICPL 20340 were subjected to protein structure modeling using the online homology modeling tool SWISS-MODEL [37]. The *TFL1* protein from *Arabidopsis thaliana* (1wko.1A) was chosen as a template for homology-based modeling because it shares 77.40% sequence identity with ICPL 20340 and 74.57% with GT 104, respectively. The quality of the modeled protein was assessed using the Global Model Quality Estimation (GMQE) and Qualitative Model Energy Analysis (QMEAN) Z-score parameters [38,39]. The GMQE score, which ranges from 0 to 1, was used to select the most reliable model. A higher number indicates greater dependability. The QMEAN Z-score estimates the “degree of nativeness” of the structural features found in the model on a global scale. A QMEAN Z score of zero indicate that the model and experimental structures agree well. Models with a score of −4.0 or less are considered low quality. PROCHECK was used to monitor the quality of the resulting models using Ramachandran plot analysis [40]. The UCSF Chimera package [41] was used to create the molecular graphics images. The predicted protein sequences of GT 104 and ICPL 20340 were analyzed for motifs using an online tool provided by GenomeNet https://www.genome.jp/tools/motif/ (accessed on 14 June 2022). The predicted protein sequences were compared to motif libraries from the PROSITE database.

## 5. Conclusions

Allelic characterization of the loci responsible for stem architecture and floral transition may provide critical insights to decipher floral induction pathways, which will eventually open doors for applying appropriate modifications to breed desirable growth habits and photoperiod insensitivity. This is the first report on the characterization of the *CcTFL1* locus in pigeon pea and two wild relatives. An InDel discovered in the present study is accountable for determinate growth habits. This functional polymorphism can be employed to carry out deletion in the first exon through CRISPR-Cas9-mediated genome editing to manipulate growth habits in pigeon pea. Further, validation of these findings in cultivated pigeon pea and their wild relatives indicated a prevalence of InDel over a wider scale, including existing breeding and natural populations. Proteomic interventions may decipher the downstream cascades associated with phosphorylation and related post-translational modifications of protein and protein crosstalk.

## Figures and Tables

**Table 1:** Primers utilized for amplification of *CcTFL1* locus.

Primer	Sequences	Reference
Z505F	5′AGCTCACACTCCCTTTCACA3′	Designed from accession NW017984051.1
Z506R	5′GGCCACATGTGAGGATCAAT3′	
CcTFL1_f5b_F	5′GCCTCTAATAGTGGGAAGAGTC3′	[16]
CcTFL1_f5a_R	5′TTGATGTGATGAAAGGATGC3′	

F: Forward primer, R: Reverse primer.

**Table 2:** GeneMark exon prediction for *CcTFL1* in pigeon pea varieties.

Genotype		Type, Range, and Length of the Exon				Sequence Length (bp)
		Initial	Internal	Internal	Terminal	
PADT 16 ^D^	R	106–225	429–490	998–1038	1134–1351	1387
	L	120 *	62	41	218	
ICPL 20340 ^D^	R	105–224	428–489	995–1035	1131–1348	1419
	L	120 *	62	41	218	
BDN 711 ^I^	R	34–234	438–499	1007–1047	1143–1360	1387
	L	201 *	62	41	218	
Vaishali ^I^	R	34–234	438–499	1007–1047	1143–1360	1387
	L	201 *	62	41	218	
GT 104 ^I^	R	35–235	439–500	1008–1048	1144–1361	1391
	L	201 *	62	41	218	
GT 105 ^I^	R	34–234	438–499	1007–1047	1143–1360	1388
	L	201 *	62	41	218	
*C. scarabaeoides* ^I^	R	45–245	454–515	1019–1059	1155–1372	1449
	L	201 *	62	41	218	
*R. rothii* ^I^	R	37–237	424-485	999–1039	1141–1358	1435
	L	201 *	62	41	218	

R: Range, L: Length, *: Exon length difference, ^I^: IDT growth habit, ^D^: DT growth habit.

## Data Availability

The sequencing data have been submitted to the NCBI GenBank database (Accession no.: ON711014 to ON711029, OQ540751 to OQ540753) and included as Supplementary Material.

## Supplementary Materials

The following supporting information can be downloaded at: https://www.mdpi.com/article/10.3390/plants12112168/s1, The dataset supporting the conclusions of this article is included within the article. The sequencing data have been submitted to the NCBI GenBank database (Accession nos. ON711014 to ON711029, OQ540751 to OQ540753) and included as Supplementary Material.

## References

[1] Mula M.G., Saxena K.B. (2010). Lifting the Level of Awareness on Pigeonpea—A Global Perspective.

[2] Liu B., Watanabe S., Uchiyama T., Kong F., Kanazawa A., Xia Z., Nagamatsu A., Arai M., Yamada T., Kitamura K. (2010). The soybean stem growth habit gene *Dt1* is an ortholog of *Arabidopsis TERMINAL FLOWER1*. Plant Physiol..

[3] Hanzawa Y., Money T., Bradley D. (2005). A single amino acid converts a repressor to an activator of flowering. Proc. Natl. Acad. Sci. USA.

[4] Wickland D.P., Hanzawa Y. (2015). The *FLOWERING LOCUS T/TERMINAL FLOWER 1* gene family: Functional evolution and molecular mechanisms. Mol. Plant.

[5] Koinange E.M.K., Singh P., Gept P. (1996). Genetic control of the domestication syndrome in common bean. Crop Sci..

[6] Ramtekey V., Bhuriya A., Ayer D., Parekh V., Modha K., Kale B., Vadodariya G., Patel R. (2019). Molecular tagging of photoperiod responsive flowering in Indian bean [*Lablab purpureus* (L.) Sweet]. Indian J. Genet. Plant Breed..

[7] Modha K., Kale B., Borwal D., Ramtekey V., Arpit B. (2019). Inheritance pattern of photoperiod responsive flowering, growth habit and flower colour in Indian bean [*Lablab purpureus* (L.) Sweet.]. Electron. J. Plant Breed..

[8] Ahn J.H., Miller D., Winter V.J., Banfield M.J., Lee J.H., Yoo S.Y., Henz S.R., Brady R.L., Weigel D. (2006). A divergent external loop confers antagonistic activity on floral regulators *FT* and *TFL1*. EMBO J..

[9] Kapoor R.K., Gupta S.C. (1991). Inheritance of growth habit in pigeonpea. Crop. Sci..

[10] Bradley D., Ratcliffe O., Vincent C., Carpenter R., Coen E. (1997). Inflorescence commitment and architecture in *Arabidopsis*. Science.

[11] Pnueli L., Gutfinger T., Hareven D., Ben-Naim O., Ron N., Adir N., Lifschitz E. (2001). Tomato SP-interacting proteins define a conserved signaling system that regulates shoot architecture and flowering. Plant Cell.

[12] Benlloch R., Berbel A., Ali L., Gohari G., Millán T., Madueño F. (2015). Genetic control of inflorescence architecture in legumes. Front. Plant Sci..

[13] Foucher F., Morin J., Courtiade J., Cadioux S., Ellis N., Banfield M.J., Rameau C. (2003). *DETERMINATE* and *LATE FLOWERING* are two *TERMINAL FLOWER1/CENTRORADIALIS* homologs that control two distinct phases of flowering initiation and development in pea. Plant Cell.

[14] Kwak M., Velasco D., Gepts P. (2008). Mapping homologous sequences for determinacy and photoperiod sensitivity in common bean (*Phaseolus Vulgaris*). J. Hered..

[15] Hanano S., Goto K. (2011). *Arabidopsis TERMINAL FLOWER1* is involved in the regulation of flowering time and inflorescence development through transcriptional repression. Plant Cell.

[16] Mir R.R., Kudapa H., Srikanth S., Saxena R.K., Sharma A., Azam S., Saxena K., Varma Penmetsa R., Varshney R.K. (2014). Candidate gene analysis for determinacy in pigeonpea (*Cajanus* spp.). Theor. Appl. Genet..

[17] Saxena R.K., Obala J., Sinjushin A., Kumar C.V.S., Saxena K.B., Varshney R.K. (2017). Characterization and mapping of *Dt1* locus which co-segregates with *CcTFL1* for growth habit in pigeonpea. Theor. Appl. Genet..

[18] Iwata H., Gaston A., Remay A., Thouroude T., Jeauffre J., Kawamura K., Oyant L.H.-S., Araki T., Denoyes B., Foucher F. (2012). The *TFL1* homologue *KSN* is a regulator of continuous flowering in rose and strawberry. Plant J..

[19] Wang Y.-H., Wei K.Y., Smolke C.D. (2013). Synthetic biology: Advancing the design of diverse genetic systems. Annu. Rev. Chem. Biomol. Eng..

[20] Shannon S., Meeks-Wagner D.R. (1991). A mutation in the *Arabidopsis TFL1* gene affects inflorescence meristem development. Plant Cell.

[21] Repinski S.L., Kwak M., Gepts P. (2012). The common bean growth habit gene *PvTFL1y* is a functional homolog of *Arabidopsis TFL1*. Theor. Appl. Genet..

[22] Kaldate S., Patel A., Modha K., Parekh V., Kale B., Vadodariya G., Patel R. (2021). Allelic characterization and protein structure analysis reveals the involvement of splice site mutation for growth habit differences in *Lablab purpureus* (L.) Sweet. J. Genet. Eng. Biotechnol..

[23] Tian Z., Wang X., Lee R., Li Y., Specht J.E., Nelson R.L., McClean P.E., Qiu L., Ma J. (2010). Artificial selection for determinate growth habit in soybean. Proc. Natl. Acad. Sci. USA.

[24] Wang Y.-H., He X.-H., Yu H.-X., Mo X., Fan Y., Fan Z.-Y., Xie X.-J., Liu Y., Luo C. (2021). Overexpression of four *MiTFL1* genes from mango delays the flowering time in transgenic *Arabidopsis*. BMC Plant Biol..

[25] Dhanasekar P., Reddy K.S. (2015). A novel mutation in *TFL1* homolog affecting determinacy in cowpea (*Vigna unguiculata*). Mol. Genet. Genom..

[26] Kishimoto A., Nishiyama K., Nakanishi H., Uratsuji Y., Nomura H., Takeyama Y., Nishizuka Y. (1985). Studies on the phosphorylation of myelin basic protein by protein kinase C and adenosine 3′:5′-monophosphate-dependent protein kinase. J. Biol. Chem..

[27] Simeunovic A., Mair A., Wurzinger B., Teige M. (2016). Know where your clients are: Subcellular localization and targets of calcium-dependent protein kinases. J. Exp. Bot..

[28] Xi L., Zhang Z., Herold S., Kassem S., Wu X.N., Schulze W.X. (2021). Phosphorylation site motifs in plant protein kinases and their substrates. Methods Mol. Biol..

[29] Kawamoto N., Sasabe M., Endo M., Machida Y., Araki T. (2015). Calcium-dependent protein kinases responsible for the phosphorylation of a bZIP transcription factor *FD* crucial for the florigen complex formation. Sci. Rep..

[30] Ren Y., Li Y., Jiang Y., Wu B., Miao Y. (2017). Phosphorylation of WHIRLY1 by CIPK14 shifts its localization and dual functions in *Arabidopsis*. Mol. Plant.

[31] Goretti D., Silvestre M., Collani S., Langenecker T., Méndez C., Madueño F., Schmid M. (2020). *TERMINAL FLOWER1* functions as a mobile transcriptional cofactor in the shoot apical meristem. Plant Physiol..

[32] Taoka K., Ohki I., Tsuji H., Kojima C., Shimamoto K. (2013). Structure and Function of florigen and the receptor complex. Trends Plant Sci..

[33] Doyle J.J., Doyle J.L. (1990). Isolation of plant DNA from fresh tissue. Focus.

[34] Altschul S.F., Gish W., Miller W., Myers E.W., Lipman D.J. (1990). Basic local alignment search tool. J. Mol. Biol..

[35] Hall T.A. (1999). A user-friendly biological sequence alignment editor and analysis program for Windows 95/98/NT. Nucleic Acids Symp. Ser..

[36] Lomsadze A. (2005). Gene identification in novel eukaryotic genomes by self-training algorithm. Nucleic Acids Res..

[37] Arnold K., Bordoli L., Kopp J., Schwede T. (2006). The SWISS-MODEL workspace: A web-based environment for protein structure homology modelling. Bioinformatics.

[38] Benkert P., Tosatto S.C.E., Schwede T. (2009). Global and local model quality estimation at CASP8 using the scoring functions QMEAN and QMEANclust. Proteins Struct. Funct. Bioinform..

[39] Studer G., Rempfer C., Waterhouse A.M., Gumienny R., Haas J., Schwede T. (2020). QMEANDisCo—Distance constraints applied on model quality estimation. Bioinformatics.

[40] Laskowski R.A., MacArthur M.W., Moss D.S., Thornton J.M. (1993). PROCHECK: A program to check the stereochemical quality of protein structures. J. Appl. Crystallogr..

[41] Pettersen E.F., Goddard T.D., Huang C.C., Couch G.S., Greenblatt D.M., Meng E.C., Ferrin T.E. (2004). UCSF Chimera:A visualization system for exploratory research and analysis. J. Comput. Chem..

