# Supplementary material for: Characterization of CcTFL1 Governing Plant Architecture in Pigeon pea (Cajanus cajan (L.) Millsp.)
Source: Plants (Basel). 2023 May 30;12(11):2168. doi: 10.3390/plants12112168 (PMC10255364; doi:10.3390/plants12112168)
Supplement: Supplementary file 1 [file plants-12-02168-s001.zip › plants-2411149-supplementary.pdf]

Supplementary Information

Complete characterization and validation of *CcTFL1* governing plant architecture in pigeonpea (*Cajanus cajan* (L.) Millsp.)

Authors and affiliations:

Isha Mendapara

Department of Genetics and Plant Breeding, N. M. College of Agriculture, Navsari Agricultural University, Navsari 396 450, Gujarat, India

Kaushal Modha*

Department of Genetics and Plant Breeding, N. M. College of Agriculture, Navsari Agricultural University, Navsari 396 450, Gujarat, India

Sunayan Patel

Department of Genetics and Plant Breeding, College of Agriculture, Navsari Agricultural University Campus, Bharuch 392 012, Gujarat, India

Vipulkumar Parekh

Department of Basic Science and Humanities, College of Forestry, Navsari Agricultural University, Navsari 396 450, Gujarat, India

Ritesh Patel

Department of Genetics and Plant Breeding, N. M. College of Agriculture, Navsari Agricultural University, Navsari 396 450, Gujarat, India

Digvijay Chauhan

Pulses and Castor Research Station, Navsari Agricultural University, Navsari 396 450, Gujarat, India

Kirti Bardhan

Department of Basic Science and Humanities, College of Forestry, Navsari Agricultural University, Navsari 396 450, Gujarat, India

Manzer H Siddiqui

Department of Botany and Microbiology, College of Science, King Saud University, Riyadh, 11451, Saudi Arabia

Saud Alamri

Department of Botany and Microbiology, College of Science, King Saud University, Riyadh, 11451, Saudi Arabia

Md. Atikur Rahmane

Grassland & Forages Division, National Institute of Animal Science, Rural Development Administration, Cheonan, 330-801, Republic of Korea

>ON711024 [*Cajanus cajan*] determinate growth habit Genotype PADT 16 gene *CcTFL1* complete cds

CATTCTTCTCGCTGTCTTCCTTCTTCCTCTTATAATGGCAAGAATGCCTATAGAGCCTCTAATAGTG
GGAAGAGTCATAGGAGAAGTTCTTGATTCTTTCACCACATGACTGTGAGTTACAACAAGAAGCAA
GTCTACAATGGCCATGAGCTCTTCCCTTCCACTGTCAACACCATACCCAAGGTTGAGATTGATGGT
GGTGATATGAGGTCCTTCTTCACACTGGTATATATGTATCTCTTCTTATATTCCCTTTTCTTTGAAGA
ACAAAGGGGTGAAAAAAAAGAAAAAATTAAGCTGTTTTCCAGTGTGTTCGTTTGTTGTTTTCTGTA
ATCATGCTCACACAATCACACTATATGCCTCTTTTATTGGAGTTTTCTTCAAGAGTACTGAATCATT

AAGCTAAATGTCTCCCTTTTTTGTCACAGATCATGACAGACCCGGATGTTCCTGGCCCTAGTGATCC
TTATCTGAGAGAGCACTTGCACTGGTACTCAATTATACCATCATTTTAGTTTATTAAACATTGCTTA
AATACTTAAGCTGTAACACTAAACTAACCATTTATAACATAAAAGAAAAACCCACCACACCACAT
AGCCACTGGATTCTTTTAACAACTCAACAAAATTTTTAAGATAATCATTTAAAAGTACTTAACTGTT
TACCCATCTGACAATATTCACGTCAGATGTCTGTGTATTTTAAGCCTAATAGAAGTTTGAAGTTTTA
CAAGATATATATGCATCCTTTCATCACATCAAATTAACCACTGAAGTAAATGAAGAAGGTACATAT
ATACATACCTTTAACTGTATTCTGTAAAAGTAGCACGTTCTACCAAATCCATCCACCCTAAAACCA
AAACAATCTCAAAGGGTGAATAAATCTCGAAGTCTCAAACATAACATAATTAACGGTATATATAT
GTAAGGTAAACTAGCCATTCATAGGTATCTAACAGTCAACAAATTTGTTGGAAATATTCTTTTTGC
AGGATAGTGACAGATATTCCAGGCACAACAGATGCCACATTTGGTAGGTTCATGTAAATGATTGGT
ATATAAGGGAACTTTCAACTTACTACTGATGATGAGAGAGACTGACAAATATGATCTATCTTGCTG
TTGTAGGGAAAGAGTTGGTGAGCTATGAGATCCCAAAGCCTAATATTGGGATCCATAGGTTTGTGT
TTGTCCTGTTCAAGCAAAAGCGTAGGCAGTGTGTTACTCCACCTGCTTCAAGGGATCACTTCAACA
CACGCAATTTCGCCGCACAGAATGACCTTGGCCTCCCTGTGGCTGCTGTCTACTTCAATGCACAGA
GGGAAACGGCTGCAAGAAGACGCTAGCGAGCATACTTGGTGATTGCATGCTGCACACTCAAG

>ON711025 [*Cajanus cajan*] determinate growth habit Genotype ICPL 20340 gene *CcTFL1* complete cds

CCTTCTTCTCTCTTCTTCCTTCTTCCTCTTATAATGGCAAGAATGCCTATAGAGCCTCTAATAGTGG
GAAGAGTCATAGGAGAAGTTCTTGATTCTTTCACCACATGACTGTGAGTTACAACAAGAAGCAAG
TCTACAATGGCCATGAGCTCTTCCCTTCCACTGTCAACACCATACCCAAGGTTGAGATTGATGGTG
GTGATATGAGGTCCTTCTTCACACTGGTATATATGTATCTCTTCTTATATTCCCTTTTCTTTGAAGAA
CAAAGGGGTGAAAAAAAAGAAAAAATTAAGCTGTTTTCCAGTGTGTTCGTTTGTTGTTTTCTGTAA
TCATGCTCACACAATCACACTATATGCCTCTTTTATTGGAGTTTTCTTCAAGAGTACTGAATCATTA
AGCTAAATGTCTCCCTTTTTTGTCACAGATCATGACAGACCCGGATGTTCCTGGCCCTAGTGATCCT
TATCTGAGAGAGCACTTGCACTGGTACTCAATTATACCATCATTTTAGTTTATTAAACATTGCTTAA
ATACTTAAGCTGTAACACTAAACTAACCATTTATAACTTAAAAAAAAACCCCCCCCCCCATAGCC
CCTGGATTCTTTTAACAACTCAACAAAATTTTTAAAATAATCATTTAAAAGTACTTAACTGGTTACC
CATCTGACAATATTCACGTCAGATGTCTGTGTATTTTAAGCCTAATAGAAGTTTGAAGTTTTACAA
GATATATATGCATCCTTTCATCACATCAAATTAACCACTGAAGTAAATGAAGAAGGTACATATATA
CATACCTTTAACTGTATTCTGTAAAAGTAGCACGTTCTACCAAATCCATCCACCCTAAAACCAAAA
CAATCTCAAAGGGTGAATAAATCTCGAAGTCTCAAACATAACATAATTAACGGTATATATATGTAA
GGTAAACTAGCCATTCATAGGTATCTAACAGTCAACAAATTTGTTGGAAATATTCTTTTTGCAGGA
TAGTGACAGATATTCCAGGCACAACAGATGCCACATTTGGTAGGTTCATGTAAATGATTGGTATAT
AAGGGAACTTTCAACTTACTACTGATGATGAGAGAGACTGACAAATATGATCTATCTTGCTGTTGT
AGGGAAAGAGTTGGTGAGCTATGAGATCCCAAAGCCTAATATTGGGATCCATAGGTTTGTGTTTGT
CCTGTTCAAGCAAAAGCGTAGGCAGTGTGTTACTCCACCTGCTTCAAGGGATCACTTCAACACACG
CAATTTCGCCGCACAGAATGACCTTGGCCTCCCTGTGGCTGCTGTCTACTTCAATGCACAGAGGGA
AACGGCTGCAAGAAGACGCTAGCGAGCATACTTGGTGATTGCTTCTGCAACCAAGGTAGTATTGA
ATAAAGCATTAGGTTCCCTTCTAACCGG

>ON711026 [*Cajanus cajan*] indeterminate growth habit Genotype BDN 711 gene *CcTFL1* complete cds

CTTTCTTCTCTCTTCTTCCTTCTTCCTCTTATAATGGCAAGAATGCCTATAGAGCCTCTAATAGTGGG
AAGAGTCATAGGAGAAGTTCTTGATTCTTTCACCACAAGCATAAAAATGACTGTGAGTTACAACA
AGAAGCAAGTCTACAATGGCCATGAGCTCTTCCCTTCCACTGTCAACACCATACCCAAGGTTGAGA
TTGATGGTGGTGATATGAGGTCCTTCTTCACACTGGTATATATGTATCTCTTCTTATATTCCCTTTTC
TTTGAAGAACAAAGGGGTGAAAAAAAAGAAAAAATTAAGCTGTTTTCCAGTGTGTTCGTTTGTTGT
TTTCTGTAATCATGCTCACACAATCACACTATATGCCTCTTTTATTGGAGTTTTCTTCAAGAGTACT
GAATCATTAAGCTAAATGTCTCCCTTTTTTGTCACAGATCATGACAGACCCGGATGTTCCTGGCCCT
AGTGATCCTTATCTGAGAGAGCACTTGCACTGGTACTCAATTATACCATCATTTTAGTTTATTAAAC
ATTGCTTAAATACTTAAGCTGTAACACTAAACTAACCATTTATAACATAAAAGAAAAACCCACCAC
ACCACATAGCCACTGGATTCTTTTAACAACTCAACAAAAATTTTAAGATAATCATTTAAAAGTACT
TAACTGTTTACCCATCTGACAATATTCACGTCAGATGTCTGTGTATTTTAAGCCTAATAGAAGTTTG
AAGTTTTACAAGATATATATGCATCCTTTCATCACATCAAATTAACCACTGAAGTAAATGAAGAAG
GTACATATATACATACCTTTAACTGTATTCTGTAAAAGTAGCACGTTCTACCAAATCCATCCACCCT
AAAACCAAAACAATCTCAAAGGGTGAATAAATCTCGAAGTCTCAAACATAACATAATTAACGGTA
TATATATGTAAGGTAAACTAGCCATTCATAGGTATCTAACAGTCAACAAATTTGTTGGAAATATTC
TTTTTGCAGGATAGTGACAGATATTCCAGGCACAACAGATGCCACATTTGGTAGGTTCATGTAAAT
GATTGGTATATAAGGGAACTTTCAACTTACTACTGATGATGAGAGAGACTGACAAATATGATCTAT
CTTGCTGTTGTAGGGAAAGAGTTGGTGAGCTATGAGATCCCAAAGCCTAATATTGGGATCCATAGG
TTTGTGTTTGTCCTGTTCAAGCAAAAGCGTAGGCAGTGTGTTACTCCACCTGCTTCAAGGGATCACT
TCAACACACGCAATTTCGCCGCACAGAATGACCTTGGCCTCCCTGTGGCTGCTGTCTACTTCAATG
CACAGAGGGAAACGGCTGCAAGAAGACGCTAGCGAGCATACTTGGTGATTGCTTACTGC

>ON711027 [*Cajanus cajan*] indeterminate growth habit Genotype Vaishali gene *CcTFL1* complete cds
CCTTCTTCTCTCTTCTTCCTTCTTCCTCTTATAATGGCAAGAATGCCTATAGAGCCTCTAATAGTGG
GAAGAGTCATAGGAGAAGTTCTTGATTCTTTCACCACAAGCATAAAAATGACTGTGAGTTACAAC
AAGAAGCAAGTCTACAATGGCCATGAGCTCTTCCCTTCCACTGTCAACACCATACCCAAGGTTGAG
ATTGATGGTGGTGATATGAGGTCCTTCTTCACACTGGTATATATGTATCTCTTCTTATATTCCCTTTT
CTTTGAAGAACAAAGGGGTGAAAAAAAAGAAAAAATTAAGCTGTTTTCCAGTGTGTTCGTTTGTTG
TTTTCTGTAATCATGCTCACACAATCACACTATATGCCTCTTTTATTGGAGTTTTCTTCAAGAGTAC
TGAATCATTAAGCTAAATGTCTCCCTTTTTTGTCACAGATCATGACAGACCCGGATGTTCCTGGCCC
TAGTGATCCTTATCTGAGAGAGCACTTGCACTGGTACTCAATTATACCATCATTTTAGTTTATTAAA
CATTGCTTAGATACTTAAGCTGTAACACTAAACTAACCATTTATAACATAAAAGAGAAACCCACCA
CACCACATAGCCACTGGATTCTTTTAACAACTCAACAGAAATTTTAAGATAATCATTTAAAAGTAC
TTAACTGTTTACCCATCTGACAATATTCACGTCAGATGTCTGTGTATGTTAAGCCTAATAGAAGTTT
GAAGTTTTACAAGATATATATGCATCCTTTCATCACATCAGATTAACCACTGAAGTAAATGAAGAA
GGTACATATATACATACCTTTAACTGTATTCTGTAAAAGTAGCACGTTCTACCAAATCCATCCACC
CTAAAACCAAAACAATCTCAAAGGGTGAATAAATCTCGAAGTCTCAAACATAACATAATTAACGG
TATATATATGTAAGGTAAACTAGCCATTCATAGGTATCTAACAGTCAACAAATTTGTTGGAAATAT
TCTTTTTGCAGGATAGTGACAGATATTCCAGGCACAACAGATGCCACATTTGGTAGGTTCATGTAA
ATGATTGGTATATAAGGGAACTTTCAACTTACTACTGATGATGAGAGAGACTGACAAATATGATCT
ATCTTGCTGTTGTAGGGAAAGAGTTGGTGAGCTATGAGATCCCAAAGCCTAATATTGGGATCCATA

GGTTTGTGTTTGTCCTGTTCAAGCAAAAGCGTAGGCAGTGTGTTACTCCACCTGCTTCAAGGGATC
ACTTCAACACACGCAATTTCGCCGCACAGAATGACCTTGGCCTCCCTGTGGCTGCTGTCTACTTCA
ATGCACAGAGGGAAACGGCTGCAAGAAGACGCTAGCGAGCATACTTGGTGATTGCTTCTGCA

>ON711028 [Cajanus cajan] indeterminate growth habit Genotype GT 104 gene *CcTFL1* complete cds
GCTTCTTCTCGCTGTCTTACTTCTTCCTCTTATAATGGCAAGAATGCCTATAGAGCCTCTAATAGTG
GGAAGAGTCATAGGAGAAGTTCTTGATTCTTTCACCACAAGCATAAAAATGACTGTGAGTTACAA
CAAGAAGCAAGTCTACAATGGCCATGAGCTCTTCCCTTCCACTGTCAACACCATACCCAAGGTTGA
GATTGATGGTGGTGATATGAGGTCCTTCTTCACACTGGTATATATGTATCTCTTCTTATATTCCCTTT
TCTTTGAAGAACAAAGGGGTGAAAAAAAAGAAAAAATTAAGCTGTTTTCCAGTGTGTTCGTTTGTT
GTTTTCTGTAATCATGCTCACACAATCACACTATATGCCTCTTTTATTGGAGTTTTCTTCAAGAGTA
CTGAATCATTAAGCTAAATGTCTCCCTTTTTTGTCACAGATCATGACAGACCCGGATGTTCCTGGCC
CTAGTGATCCTTATCTGAGAGAGCACTTGCACTGGTACTCAATTATACCATCATTTTAGTTTATTAA
ACATTGCTTAAATACTTAAGCTGTAACACTAAACTAACCATTTATAACATAAAAGAAAAACCCACC
ACACCACATAGCCACTGGATTCTTTTAACAACTCAACAAAAATTTTAAGATAATCATTTAAAAGTA
CTTAACTGTTTACCCATCTGACAATATTCACGTCAGATGTCTGTGTATTTTAAGCCTAATAGAAGTT
TGAAGTTTTACAAGATATATATGCATCCTTTCATCACATCAAATTAACCACTGAAGTAAATGAAGA
AGGTACATATATACATACCTTTAACTGTATTCTGTAAAAGTAGCACGTTCTACCAAATCCATCCAC
CCTAAAACCAAAACAATCTCAAAGGGTGAATAAATCTCGAAGTCTCAAACATAACATAATTAACG
GTATATATATGTAAGGTAAACTAGCCATTCATAGGTATCTAACAGTCAACAAATTTGTTGGAAATA
TTCTTTTTGCAGGATAGTGACAGATATTCCAGGCACAACAGATGCCACATTTGGTAGGTTCATGTA
AATGATTGGTATATAAGGGAACTTTCAACTTACTACTGATGATGAGAGAGACTGACAAATATGATC
TATCTTGCTGTTGTAGGGAAAGAGTTGGTGAGCTATGAGATCCCAAAGCCTAATATTGGGATCCAT
AGGTTTGTGTTTGTCCTGTTCAAGCAAAAGCGTAGGCAGTGTGTTACTCCACCTGCTTCAAGGGAT
CACTTCAACACACGCAATTTCGCCGCACAGAATGACCTTGGCCTCCCTGTGGCTGCTGTCTACTTC
AATGCACAGAGGGAAACGGCTGCAAGAAGACGCTAGCGAGCATACTTGGTGATTGTATGCTGCAC
A

>ON711029 [Cajanus cajan] indeterminate growth habit Genotype GT 105 gene CcTFL1 complete cds
CTTTCTTCTCTCTTCTTCCTTCTTCCTCTTATAATGGCAAGAATGCCTATAGAGCCTCTAATAGTGGG
AAGAGTCATAGGAGAAGTTCTTGATTCTTTCACCACAAGCATAAAAATGACTGTGAGTTACAACA
AGAAGCAAGTCTACAATGGCCATGAGCTCTTCCCTTCCACTGTCAACACCATACCCAAGGTTGAGA
TTGATGGTGGTGATATGAGGTCCTTCTTCACACTGGTATATATGTATCTCTTCTTATATTCCCTTTTC
TTTGAAGAACAAAGGGGTGAAAAAAAAGAAAAAATTAAGCTGTTTTCCAGTGTGTTCGTTTGTTGT
TTTCTGTAATCATGCTCACACAATCACACTATATGCCTCTTTTATTGGAGTTTTCTTCAAGAGTACT
GAATCATTAAGCTAAATGTCTCCCTTTTTTGTCACAGATCATGACAGACCCGGATGTTCCTGGCCCT
AGTGATCCTTATCTGAGAGAGCACTTGCACTGGTACTCAATTATACCATCATTTTAGTTTATTAAAC
ATTGCTTAAATACTTAAGCTGTAACACTAAACTAACCATTTATAACATAAAAGAAAAACCCACCAC
ACCACATAGCCACTGGATTCTTTTAACAACTCAACAAAAATTTTAAGATAATCATTTAAAAGTACT
TAACTGTTTACCCATCTGACAATATTCACGTCAGATGTCTGTGTATTTTAAGCCTAATAGAAGTTTG
AAGTTTTACAAGATATATATGCATCCTTTCATCACATCAAATTAACCACTGAAGTAAATGAAGAAG

GTACATATATACATACCTTTAACTGTATTCTGTAAAAGTAGCACGTTCTACCAAATCCATCCACCCT
AAAACCAAAACAATCTCAAAGGGTGAATAAATCTCGAAGTCTCAAACATAACATAATTAACGGTA
TATATATGTAAGGTAAACTAGCCATTCATAGGTATCTAACAGTCAACAAATTTGTTGGAAATATTC
TTTTTGCAGGATAGTGACAGATATTCCAGGCACAACAGATGCCACATTTGGTAGGTTCATGTAAAT
GATTGGTATATAAGGGAACTTTCAACTTACTACTGATGATGAGAGAGACTGACAAATATGATCTAT
CTTGCTGTTGTAGGGAAAGAGTTGGTGAGCTATGAGATCCCAAAGCCTAATATTGGGATCCATAGG
TTTGTGTTTGTCCTGTTCAAGCAAAAGCGTAGGCAGTGTGTTACTCCACCTGCTTCAAGGGATCACT
TCAACACACGCAATTTCGCCGCACAGAATGACCTTGGCCTCCCTGTGGCTGCTGTCTACTTCAATG
CACAGAGGGAAACGGCTGCAAGAAGACGCTAGCGAGCATACTTGGTGATTGCTTCTGCAC

>ON711014 [Cajanus cajan] determinate growth habit Genotype GT 100 gene CcTFL1 partial cds
CCACATGACTGTGAGTTACAACAAGAAGCAAGTCTACAATGGCCATGAGCTCTTCCCTTCCACTGT
CAACACCATACCCAAGGTTGAGATTGATGGTGGTGATATGAGGTCCTTCTTCACACTGGTATATAT
GTATCTCTTCTTATATTCCCTTTTCTTTGAAGAACAAAGGGGTGAAAAAAAAGAAAAAATTAAGCT
GTTTTCCAGTGTGTTCGTTTGTTGTTTTCTGTAATCATGCTCACACAATCACACTATATGCCTCTTTT
ATTGGAGTTTTCTTCAAGAGTACTGAATCATTAAGCTAAATGTCTCCCTTTTTTGTCACAGATCATG
ACAGACCCGGATGTTCCTGGCCCTAGTGATCCTTATCTGAGAGAGCACTTGCACTGGTACTCAATT
ATACCATCATTTTAGTTTATTAAACATTGCTTAAATACTTAAGCTGTAACACTAAACTAACCATTTA
TAACATAAAAGAAAAACCCACCACACCACATAGCCACTGGATTCTTTTAACAACTCAACAAAATTT
TTAAGATAATCATTTAAAAGTACTTAACTGTTTACCCATCTGACAATATTCACGTCAGATGTCTGTG
TATTTTAAGCCTAATAGAAGTTTGAAGTTTTACAAGATATATATGCATCCTTTCATCACATCAAAA
A

> ON711015 [Cajanus cajan] determinate growth habit Genotype ICPL 87 gene CcTFL1 partial cds
CCACATGACTGTGAGTTACAACAAGAAGCAAGTCTACAATGGCCATGAGCTCTTCCCTTCCACTGT
CAACACCATACCCAAGGTTGAGATTGATGGTGGTGATATGAGGTCCTTCTTCACACTGGTATATAT
GTATCTCTTCTTATATTCCCTTTTCTTTGAAGAACAAAGGGGTGAAAAAAAAGAAAAAATTAAGCT
GTTTTCCAGTGTGTTCGTTTGTTGTTTTCTGTAATCATGCTCACACAATCACACTATATGCCTCTTTT
ATTGGAGTTTTCTTCAAGAGTACTGAATCATTAAGCTAAATGTCTCCCTTTTTTGTCACAGATCATG
ACAGACCCGGATGTTCCTGGCCCTAGTGATCCTTATCTGAGAGAGCACTTGCACTGGTACTCAATT
ATACCATCATTTTAGTTTATTAAACATTGCTTAAATACTTAAGCTGTAACACTAAACTAACCATTTA
TAACATAAAAGAAAAACCCACCACACCACATAGCCACTGGATTCTTTTAACAACTCAACAAAATTT
TTAAGATAATCATTTAAAAGTACTTAACTGTTTACCCATCTGACAATATTCACGTCAGATGTCTGTG
TATTTTAAGCCTAATAGAAGTTTGAAGTTTTACAAGATATATATGCATCCTTTCATCAACATCAAA
AA

> ON711016 [Cajanus cajan] determinate growth habit Genotype AVPP 1 gene CcTFL1 partial cds
CCACATGACTGTGAGTTACAACAAGAAGCAAGTCTACAATGGCCATGAGCTCTTCCCTTCCACTGT
CAACACCATACCCAAGGTTGAGATTGATGGTGGTGATATGAGGTCCTTCTTCACACTGGTATATAT
GTATCTCTTCTTATATTCCCTTTTCTTTGAAGAACAAAGGGGTGAAAAAAAAGAAAAAATTAAGCT
GTTTTCCAGTGTGTTCGTTTGTTGTTTTCTGTAATCATGCTCACACAATCACACTATATGCCTCTTTT

ATTGGAGTTTTCTTCAAGAGTACTGAATCATTAAGCTAAATGTCTCCCTTTTTTGTCACAGATCATG
ACAGACCCGGATGTTCCTGGCCCTAGTGATCCTTATCTGAGAGAGCACTTGCACTGGTACTCAATT
ATACCATCATTTTAGTTTATTAAACATTGCTTAAATACTTAAGCTGTAACACTAAACTAACCATTTA
TAACATAAAAGAAAAACCCACCACACCACATAGCCACTGGATTCTTTTAACAACTCAACAAAATTT
TTAAGATAATCATTTAAAAGTACTTAACTGTTTACCCATCTGACAATATTCACGTCAGATGTCTGTG
TATTTTAAGCCTAATAGAAGTTTGAAGTTTTACAAGATATATATGCATCCTTTCATTCCACATCAAA
AG

> ON711017 [Cajanus cajan] determinate growth habit Genotype ICPL 20336 gene CcTFL1 partial cds
CCACATGACTGTGAGTTACAACAAGAAGCAAGTCTACAATGGCCATGAGCTCTTCCCTTCCACTGT
CAACACCATACCCAAGGTTGAGATTGATGGTGGTGATATGAGGTCCTTCTTCACACTGGTATATAT
GTATCTCTTCTTATATTCCCTTTTCTTTGAAGAACAAAGGGGTGAAAAAAAAGAAAAAATTAAGCT
GTTTTCCAGTGTGTTCGTTTGTTGTTTTCTGTAATCATGCTCACACAATCACACTATATGCCTCTTTT
ATTGGAGTTTTCTTCAAGAGTACTGAATCATTAAGCTAAATGTCTCCCTTTTTTGTCACAGATCATG
ACAGACCCGGATGTTCCTGGCCCTAGTGATCCTTATCTGAGAGAGCACTTGCACTGGTACTCAATT
ATACCATCATTTTAGTTTATTAAACATTGCTTAAATACTTAAGCTGTAACACTAAACTAACCATTTA
TAACATAAAAGAAAAACCCACCACACCACATAGCCACTGGATTCTTTTAACAACTCAACAAAATTT
TTAAGATAATCATTTAAAAGTACTTAACTGTTTACCCATCTGACAATATTCACGTCAGATGTCTGTG
TATTTTAAGCCTAATAGAAGTTTGAAGTTTTACAAGATATATATGCATCCTTCATTCCCATCCAAAA
A

> ON711018 [Cajanus cajan] determinate growth habit Genotype ICPL 11258 gene CcTFL1 partial cds
CCACATGACTGTGAGTTACAACAAGAAGCAAGTCTACAATGGCCATGAGCTCTTCCCTTCCACTGT
CAACACCATACCCAAGGTTGAGATTGATGGTGGTGATATGAGGTCCTTCTTCACACTGGTATATAT
GTATCTCTTCTTATATTCCCTTTTCTTTGAAGAACAAAGGGGTGAAAAAAAAGAAAAAATTAAGCT
GTTTTCCAGTGTGTTCGTTTGTTGTTTTCTGTAATCATGCTCACACAATCACACTATATGCCTCTTTT
ATTGGAGTTTTCTTCAAGAGTACTGAATCATTAAGCTAAATGTCTCCCTTTTTTGTCACAGATCATG
ACAGACCCGGATGTTCCTGGCCCTAGTGATCCTTATCTGAGAGAGCACTTGCACTGGTACTCAATT
ATACCATCATTTTAGTTTATTAAACATTGCTTAAATACTTAAGCTGTAACACTAAACTAACCATTTA
TAACATAAAAGAAAAACCCACCACACCACATAGCCACTGGATTCTTTTAACAACTCAACAAAATTT
TTAAGATAATCATTTAAAAGTACTTAACTGTTTACCCATCTGACAATATTCACGTCAGATGTCTGTG
TATTTTAAGCCTAATAGAAGTTTGAAGTTTTACAAGATATATATGCATCCTTTCATTCCACATCTAA
AAAGAA

> ON711019 [Cajanus cajan] indeterminate growth habit Genotype GT 103 gene CcTFL1 partial cds
CCACAAGCATAAAAATGACTGTGAGTTACAACAAGAAGCAAGTCTACAATGGCCATGAGCTCTTC
CCTTCCACTGTCAACACCATACCCAAGGTTGAGATTGATGGTGGTGATATGAGGTCCTTCTTCACA
CTGGTATATATGTATCTCTTCTTATATTCCCTTTTCTTTGAAGAACAAAGGGGTGAAAAAAAAGAA
AAAATTAAGCTGTTTTCCAGTGTGTTCGTTTGTTGTTTTCTGTAATCATGCTCACACAATCACACTA
TATGCCTCTTTTATTGGAGTTTTCTTCAAGAGTACTGAATCATTAAGCTAAATGTCTCCCTTTTTTGT
CACAGATCATGACAGACCCGGATGTTCCTGGCCCTAGTGATCCTTATCTGAGAGAGCACTTGCACT

GGTACTCAATTATACCATCATTTTAGTTTATTAAACATTGCTTAAATACTTAAGCTGTAACACTAAA
CTAACCATTTATAACATAAAAGAAAAACCCACCACACCACATAGCCACTGGATTCTTTTAACAACT
CAACAAAAATTTTAAGATAATCATTTAAAAGTACTTAACTGTTTACCCATCTGACAATATTCACGT
CAGATGTCTGTGTATTTTAAGCCTAATAGAAGTTTGAAGTTTTACAAGATATATATGCATCCTTTCA
TTCACATCAAAAA

> ON711020 [Cajanus cajan] indeterminate growth habit Genotype GT 101 gene CcTFL1 partial cds
CCACAAGCATAAAAATGACTGTGAGTTACAACAAGAAGCAAGTCTACAATGGCCATGAGCTCTTC
CCTTCCACTGTCAACACCATACCCAAGGTTGAGATTGATGGTGGTGATATGAGGTCCTTCTTCACA
CTGGTATATATGTATCTCTTCTTATATTCCCTTTTCTTTGAAGAACAAAGGGGTGAAAAAAAAGAA
AAAATTAAGCTGTTTTCCAGTGTGTTCGTTTGTTGTTTTCTGTAATCATGCTCACACAATCACACTA
TATGCCTCTTTTATTGGAGTTTTCTTCAAGAGTACTGAATCATTAAGCTAAATGTCTCCCTTTTTTGT
CACAGATCATGACAGACCCGGATGTTCCTGGCCCTAGTGATCCTTATCTGAGAGAGCACTTGCACT
GGTACTCAATTATACCATCATTTTAGTTTATTAAACATTGCTTAAATACTTAAGCTGTAACACTAAA
CTAACCATTTATAACATAAAAGAAAAACCCACCACACCACATAGCCACTGGATTCTTTTAACAACT
CAACAAAAATTTTAAGATAATCATTTAAAAGTACTTAACTGTTTACCCATCTGACAATATTCACGT
CAGATGTCTGTGTATTTTAAGCCTAATAGAAGTTTGAAGTTTTACAAGATATATATGCATCCTTCAT
TCCCCATCAAAAATTAAAAA

> ON711021 [Cajanus cajan] indeterminate growth habit Genotype P 992 gene CcTFL1 partial cds
CCACAAGCATAAAAATGACTGTGAGTTACAACAAGAAGCAAGTCTACAATGGCCATGAGCTCTTC
CCTTCCACTGTCAACACCATACCCAAGGTTGAGATTGATGGTGGTGATATGAGGTCCTTCTTCACA
CTGGTATATATGTATCTCTTCTTATATTCCCTTTTCTTTGAAGAACAAAGGGGTGAAAAAAAAGAA
AAAATTAAGCTGTTTTCCAGTGTGTTCGTTTGTTGTTTTCTGTAATCATGCTCACACAATCACACTA
TATGCCTCTTTTATTGGAGTTTTCTTCAAGAGTACTGAATCATTAAGCTAAATGTCTCCCTTTTTTGT
CACAGATCATGACAGACCCGGATGTTCCTGGCCCTAGTGATCCTTATCTGAGAGAGCACTTGCACT
GGTACTCAATTATACCATCATTTTAGTTTATTAAACATTGCTTAAATACTTAAGCTGTAACACTAAA
CTAACCATTTATAACATAAAAGAAAAACCCACCACACCACATAGCCACTGGATTCTTTTAACAACT
CAACAAAAATTTTAAGATAATCATTTAAAAGTACTTAACTGTTTACCCATCTGAAAATATTCACGT
CAGATGTCTGTGTATTTTAAGCCTAATAGAAGTTTGAAGTTTTACAAGATATATATGCATCC

> ON711022 [Cajanus cajan] indeterminate growth habit Genotype AGT 2 gene CcTFL1 partial cds
CCACAAGCATAAAAATGACTGTGAGTTACAACAAGAAGCAAGTCTACAATGGCCATGAGCTCTTC
CCTTCCACTGTCAACACCATACCCAAGGTTGAGATTGATGGTGGTGATATGAGGTCCTTCTTCACA
CTGGTATATATGTATCTCTTCTTATATTCCCTTTTCTTTGAAGAACAAAGGGGTGAAAAAAAAGAA
AAAATTAAGCTGTTTTCCAGTGTGTTCGTTTGTTGTTTTCTGTAATCATGCTCACACAATCACACTA
TATGCCTCTTTTATTGGAGTTTTCTTCAAGAGTACTGAATCATTAAGCTAAATGTCTCCCTTTTTTGT
CACAGATCATGACAGACCCGGATGTTCCTGGCCCTAGTGATCCTTATCTGAGAGAGCACTTGCACT
GGTACTCAATTATACCATCATTTTAGTTTATTAAACATTGCTTAAATACTTAAGCTGTAACACTAAA
CTAACCATTTATAACATAAAAGAAAAACCCACCACACCACATAGCCACTGGATTCTTTTAACAACT
CAACAAAAATTTTAAGATAATCATTTAAAAGTACTTAACTGTTTACCCATCTGACAATATTCACGT

CAGATGTCTGTGTATTTTAAGCCTAATAAAAGTTTGAAGTTTTACAAGATATATATGCATCCTTTCT
TCCCATCAAAAGCGGA

> ON711023 [Cajanus cajan] indeterminate growth habit Genotype UPAS 120 gene CcTFL1 partial cds
CCACAAGCATAAAAATGACTGTGAGTTACAACAAGAAGCAAGTCTACAATGGCCATGAGCTCTTC
CCTTCCACTGTCAACACCATACCCAAGGTTGAGATTGATGGTGGTGATATGAGGTCCTTCTTCACA
CTGGTATATATGTATCTCTTCTTATATTCCCTTTTCTTTGAAGAACAAAGGGGTGAAAAAAAAGAA
AAAATTAAGCTGTTTTCCAGTGTGTTCGTTTGTTGTTTTCTGTAATCATGCTCACACAATCACACTA
TATGCCTCTTTTATTGGAGTTTTCTTCAAGAGTACTGAATCATTAAGCTAAATGTCTCCCTTTTTTGT
CACAGATCATGACAGACCCGGATGTTCCTGGCCCTAGTGATCCTTATCTGAGAGAGCACTTGCACT
GGTACTCAATTATACCATCATTTTAGTTTATTAAACATTGCTTAAATACTTAAGCTGTAACACTAAA
CTAACCATTTATAACATAAAAGAAAAACCCACCACACCACATAGCCACTGGATTCTTTTAACAACT
CAACAAAAATTTTAAAATAATCATTTAAAAGTACTTAACTGTTTACCCATCTGACAATATTCACGT
CAGATGTCTGTGTATTTTAAGCCTAATAGAAG

>OQ540751 [Cajanus cajanifolius] indeterminate growth habit gene CcTFL1 partial cds
TTCTCTCTTCTTCCTTCTTCCTCTTATAATGGCAAGAATGCCTATAGAGCCTCTAATAGTGGGAAGA
GTCATAGGAGAAGTTCTTGATTCTTTCACCACAAGCATAAAAATGACTGTGAGTTACAACAAGAA
GCAAGTCTACAATGGCCATGAGCTCTTCCCTTCCACTGTCAACACCGTACCCAAGGTTGAGATTGA
TGGTGGTGATATGAGGTCCTTCTTCACACTGGTATATATGTATCTCTTCTTACTCCCTTTTCTTTGAA
GAACAAAAGGGTGGAAAAAAAGAAAAAATTAACTTGAGCTGTTTTCCAGTGTGTTGTTTGTTTGTT
GTTTTCTGTAATCATGCTCACACAATCACACTATATGCCTCTTTTATTGGAGTTTTCTTCAAGAGTA
CTGAATCATTAAGCTAAATGTCTCCCTTTTTTGTCACAGATCATGACAGACCCGGATGTTCCTGGCC
CTAGTGATCCTTATCTGAGAGAGCACTTGCACTGGTACTCAATTATACCATCATTTTAGTTTATTAA
ACATTGCTTAAATACTTAAGCTGTAACACTAAAATAACCATTTATAACATAAAAGAAAAACCCACC
ACACCACATAGCCACTGGATTCTTTTAACAACTCAACAAAAATTTTAAGATAATCATTTAAAAGTA
CTTAACTGTTTACCCATCTGACAATATTCACGTCAGATGTCTGTGTATTTTAAGCCTAATAGAAGTT
TTGAAGTTTTACAAGATATATATGCATCCTTTCATCACATCAAATTAACCACTGAAGTAAATGAAG
AAGGTACATATATACATACCTTTAATTGTATTCTGTAAAAGTAGCACGTTCTACCAAATCCATCCA
CCCTAAAACCAAAACAATCTCAAAGGGTGAATAAATCTCGAAGTCTCAAACATAACATAATTAAC
GGTATATATATGTAAGGTAAACTAGCCATTCCTAGGTATCTAACAGTCAACAAATTTGTTGGAAAT
ATTCTTTTTGCAGGATAGTGACAGATATTCCAGGCACAACAGATGCCACATTTGGTAGGTTCATGT
AAATGATTGGGATATAAAGGGAACTTTTCAACTTACTACGGATGATGAAGAAAAACT

>OQ540752 [Cajanus scarabaeoides] indeterminate growth habit gene CsTFL1 complete cds
AATTAAGCCTTCTTTCTCCTCCTTCCTTCCTTCTTCCTCTTATAATGGCAAGAATGCCTATAGAGCCT
CTAATAGTGGGAAGAGTCATAGGAGAAGTTCTTGATTCTTTCACCACAAGCATAAAAATGACTGTG
AGTTACAACAAGAAGCAAGTCTACAATGGCCATGAGCTCTTCCCTTCCACTGTCAACACCATACCC
AAGGTTGAGATTGATGGTGGTGATATGAGGTCCTTCTTCACACTGGTATATATGTATCTCTTCTTAT
ATTCCCTTTTCTTTGAAGAACAAACGGGTGGAAAAAAAAAGAAAAAATTAACTTGAGCTGTTTTCC
AGTGTGTTTGTTTGTTGTTTTCTGTAATCATGCTCACACAATCACACTATATGCCTTTTATTGGAGTT

TTCTTCAAGAGTACTGAATCATTAAGCTAAATGTCTCCCTTTTTTGTCACAGATCATGACAGACCCG
GATGTTCCTGGCCCTAGTGATCCTTATCTGAGAGAGCACTTGCACTGGTACTCAATTATACCATCAT
TTTAGTTTATTAAACATTGCTTAAATACTTAAGCTGTAACACTAAACTAACCATTTATAACATAAA
AGAAAAACCCACCACACCACATAGCCACTGGATTCTTTGAACAACTCAACAAAAATTTTAAGATA
ATCATTTAAAAGTACTTAACTGTTTACCCATCTGACAATATTCACGTCAGATGTCTGTGTATTTTAA
GCCTAATAGAAGTTTGAAGTTTTACAAGATATATATGCATCCTTTCATCACATCAAATTAACCACT
GAAGTAAATGAAGAAGGTACATATATACATACCTTTAATTGTATTCTGTAAAAGTAGCACGTTCTA
CCAAATCCATCCACCCTAAAACCAAAACAATCTCAAAGGGTGAATAAATCTCGAAGTCTCAAACA
TAATATAATTAACGGTATATGTAAGGTAAACTAGCCATTCATAGGTATCTAACAGTCAACAAATTT
GTTGGAAATATTCTTTTTGCAGGATAGTGACAGATATTCCAGGCACAACAGATGCCACATTTGGTA
GGTTCATGTAAATGATTGGTATATAAGGGAACTTTCAACTTACTACTGATGATGAGTGAGACTGAC
AAATATGATCTATCTTGCTGTTGTAGGGAAAGAGTTGGTGAGCTATGAGATCCCAAAGCCTAATAT
TGGGATCCATAGGTTTGTGTTTGTCCTGTTCAAGCAAAAGCGTAGGCAGTGTGTTACTCCACCTAC
TTCAAGGGATCACTTCAATACACGCAATTTCGCCGCACAGAATGACCTTGGCCTCCCTGTGGCTGC
TGTCTACTTCAATGCACAGAGGGAAACGGCTGCAAGAAGACGCTAGCGAGCATACTTGGTGTTTG
CTTCTGCAACCAAGGTAGTATTGAATAAAGCATTAGCGCAAACTGCGTTGCAGGCGGT

>OQ540753 [Rhynchosia rothii] indeterminate growth habit gene RrTFL1 complete cds
AAGCCTTCTTCTTTCTTCTTCCTTCTTCCTCTTATAATGGCAAGAATGCCTTTAGAGCCTCTAATAGT
GGGGAGAGTCATAGGAGAAGTTCTTGATTCTTTCACCACAAGCATAAAAATGACTGTGAGTTACA
ATAAGAAGCAAGTCTACAATGGCCATGAGCTCTTCCCTTCCACTGTCAACACCAAACCCAAGGTTG
AGATTGATGGTGGTGATATGAGGTCCTTCTTCACACTGGTATATATATCTCTTCTTGTTCCTTTTTCT
TTGAAGAACAAATAGGTGGAACTAAAAAATTATAAAAAAAAAACTTGAGATGTTTCCAGTGTGTT
TCTTTGTTGTTTTCTGTAATCATGTTCACATAATCCCACTATATGCCTCTTTCATTGGAGTAAAGCTA
AATGTCTCCCTTTTTTATCGCAGATCATGACAGACCCGGATGTTCCTGGCCCTAGTGATCCTTATCT
GAGAGAGCACTTGCACTGGTACTCAATATATAGATGATGCCATCATTTTAGTTTATTAAACATGCT
TAAATACCATCAAAACATAAAAGAAAAACCCACCACACCATATAGCCACCGAATTCTCTTAACAA
TTCAACAAAAAATTTATGATAATCATTTAAAAGTATTTAACTGTTTACCCATCTGACAATATCCAC
GTCAGATGTCTGTATTTTAAGCCTAATATAGAAGTTTGAAGTTTTGCAAGATATATATATATATATA
TATATATGCATTCCTCTCATCGCTTCATATTAACCACCGAGGTAAATGAAGAAGGTACATATATAC
ATACCTTTAATTGTAGTCTGTATAAGTAGCATGTTCTACCAAATCCATCCACCAAAATCAAAACAA
TCTCAAAGGGCGAATAAATCTCGAAGTCTCAAACATAACATTATTAACCGTGTGTGTGTATATTTA
TAAGGTAAACTAGCCATTCATATAGGTATCTAACAATCAACAAATTTGTTGAAAATATTCTTTTTG
CAGGATAGTGACAGATATTCCAGGCACAACAGATGCCACATTTGGTAGGTTCATGTAAATGATTAC
AAAAATGTATATATATATAAGGGAACTTACTAGTGATGATGAGAGAGACTGACAAATATGATGTA
TCTTGTTGTTGTAGGGAAAGAGTTGGTGAGCTATGAGATCCCAAAGCCTAATATTGGGATCCATAG
GTTTGTGTTTGTCCTGTTCAAGCAAAAGCGTAGGCAGTGTGTTAATCCACCTACTTCAAGGGATCA
TTTCAACACACGCAATTTCGCAGCACAGAATGACCTTGGCCTCCCTGTGGCTGCTGTCTACTTCAAT
GCACAGAGGGAAACGGCTGCAAGAAGACGCTAGCGAGCAATACTTCCTTGGTGCTTGCTTCTGCA
ACCAAGCTAGTATTGAATAAAGCGATGAGTACAAAGCCTGACGGA
